# Medical and Philosophical Intelligence

**Published:** 1810-12

**Authors:** 


### MEDICAL AND PHILOSOPHICAL INTELLIGENCE.

PROFESSOR IGNATIUS COLLA has related in a recent number of the Journal of the Medico-chirurgical Society of Parma, two cases of Tænia, which were cured by half a drachm of the fresh powder of cannabina (Eupatorium *Arabum* Pharm. Paris. Agrimonia Linn. Hemp Agrimony], and an emeto-cathartic, prescribed for obstinate indigestion. Eupatorium or agrimony* is now scarcely known as a medicine in this country, but was formerly in great esteem in several complaints, particularly in cholic, gout, and jaundice; but we believe its efficacy in Tænia has not been noticed by former writers. Sir John Hill observes, "in the worst degree of the jaundice, and under its most dreadful appearances, the greatest dependence may be placed upon the celebrated herb agrimony." Its properties are well described by Dr. Baylis, in his "Compleat Body of Practical Botanic Physic."

The operation for Empyema has latelybeen successfully performed at the Hospital at Gottingen, in a case of Hydrothorax. On the sixth of August 1808, a man, aged 23 years, was brought to the hospital at Gottingen, and placed under the care of Professor Himly. He had been previously affected with fever. After his admission, his feet became œdematous, his face slightly swelled, and his respiration difficult. The œdema soon became general, and the dyspnœa very distressing; the patient could not lie on his left side without being threatened with immediate suffocation. The complaint was supposed to be hydrothorax, and the treatment usually pursued in that complaint was vigorously put in practice, but without effect. On the 30th of August, the patient being at the point of suffocation, it was determined to perform the operation of empyema. The incision was accordingly made on the right side, between the fifth and sixth true ribs. A considerable quantity of fluid escaped through the orifice, and as it flowed, the respiration became easier. On the 31st, serum continued to flow abundantly from the wound; the patient felt much relieved, the œdema sensibly diminished, respiration became almost natural, and there was little fever. On the 19th of September, the symptoms of hydrothorax seeming to return, the wound was opened again, and about an ounce and a half of water was drawn off in the space of a minute. The patient was immediately relieved, and on the second of November quitted the hospital perfectly recovered.

The particulars of this interesting case were communicated by Dr. A. Schonberg, to A. L. M. Lullier, D. M. P.

* This is not the plant commonly known as Agrimony (*Agrimonia Eupatoria.*—LINN.) but is the *Eupatorium cannabinum,* (class *Syngenasia æqualis.*) Vernacular—Hemp Agrimony; Dutch Agrimony; Water Agrimony; Water Hemp; common Hempweed; banks of rivers and brooks; flowers in July and August; perennial. It is a common plant by the sides of ditches in the fens of Cambridgeshire. An infusion of a handful of it vomits and purges smartly. An ounce of the root in decoction is a full dose. In smaller doses the Dutch peasants take it as an alterative and anti-scorbutic. Goats eat it. Cows, horses, sheep and swine refuse it. We have added this explanatory note, that should any of our readers be disposed to employ it, they may not take for it the *inert* plant. Agrimony, so well known, for its fancied medicinal properties, and growing in every upland pasture.—EDITOR.

Hufeland's Journal of Medicine and Surgery for 1808, contains a case of numerous small worms being found in the urine of a child aged three years. It had previously passed several by stool. Dumonçeau has stated a similar fact: and Dr. Kuhn has published a dissertation entitled, Dissert. de Ascaridibus per Urinam emissis, Jena 1798.

The same Journal contains an account of an imperforate anus. A male infant was born with an obstruction of the anus, occasioned by a prolongation of the skin, in the form of a band, which extended from the coccyx to the raphè, and passed over the anus to which it closely adhered. A small hole, however, was observed on each side, directed towards the rectum, and on pressing the abdomen, a serous fluid and gas passed through one of them. On the sixth day, from the convulsive cries of the infant, and the retention of the mæconium, the malconformation was discovered. Tension and hardness of the belly, redness of the face, violent hiccups, and frequent vomiting, indicated extreme danger, when M. Moucelot, by a simple operation, cleared the external orifice of the anus, and gave vent to the matter accumulated in the rectum. The alarming symptoms soon ceased, and at the end of three days the child was entirely well.

---

### COW-POCK INSTITUTION, Dublin,

UNDER THE PATRONAGE OF

*His Grace the Lord Lieutenant, No. 55, Sackville-Street.*

Opened on the 14th of January, 1804, under the Direction of the undersigned Physicians and Surgeons of this City, for the purposes of securing a succession of Cow-Pock Matter, by inoculating gratuitously the Children of the Poor, and of supplying the different parts of the Kingdom with genuine Infection.

---

### DIRECTORS.

| *Physicians.* | *Surgeons.* |
|---|---|
| JOSEPH CLARKE, | GEORGE STEWART, |
| JAMES CLEGHORN, | RALPH S. OBRE, |
| THOMAS EVORY, | SOLOMON RICHARDS. |

An Abstract from the Register of Inoculations and Distribution of Matter.

| | Patients Inoculated. | Packets issued to Practitioners in general | Packets to Army Surgeons. |
|---|---|---|---|
| 1804 | 578 | 776 | 236 |
| 1805 | 1,032 | 1,124 | 178 |
| 1806 | 1,356 | 1,340 | 220 |
| 1807 | 2,156 | 1,790 | 320 |
| 1808 | 3,002 | 2,285 | 333 |
| 1809 | 3,941 | 2,540 | 244 |
| *Totals.* | 12,065 | 9,855 | 1,537 |

The Directors of the Institution have great pleasure in observing the progressive increase of *Vaccine Inoculation,* and the influence of experience in satisfying the Public of its efficacy. Most of the above 12,065 patients being confined to a city, where Small-Pox has been in general prevalent, must have been exposed in every possible way to its infection, by living in the same house, or frequently sleeping in the same bed with the infected. The anxiety of parents too, has often led them intentionally to expose their children to Small Pox infection. As far, however, as the immediate observation of the Institution extends, Cow-Pock has been found to resist all such trials, with three exceptions only.

It now appears by increasing experience, that in *a very few instances,* the vaccine infection will form fairly on the arm, and go through its regular stages, without being absorbed into the blood. The same thing has repeatedly happened in inoculating for the Small-Pox, where no eruptive fever or eruption succeeded the inoculation. In the three cases of Small-Pox which have succeeded vaccination, the disease has been mild and of short duration.

The efficacy of Cow-Pock, as far as Dublin is concerned, does not rest upon the proofs adduced in its favour by this Institution, for it has been extensively practised during the last five or six years. There are grounds for believing that the number vaccinated throughout the city, including the above 12,065, does not fall short of 35,000. The cases of Small-Pox following Cow-Pock, which have been reported, upon any reasonable authority, to the Institution, do not exceed six. No one who is acquainted with the careless and inattentive manner in which many practitioners have hitherto conducted vaccination, can be surprized to hear of cases of failure. The neglect of parents also to have their children examined at the regular periods after inoculation, tends to bring the practice into disrepute. To obviate this inconvenience it has been the practice for some time at this Institution, to oblige parents to deposit a small sum, to be returned after the child has gone through the disease, provided they have attended agreeably to instructions, otherwise the sum is forfeited. This regulation has had the desired effect.

It was reported at an early period of the practice, that vaccination afforded only a temporary security, which was at first limited to three years. Numerous experiments, tried in different quarters satisfactorily proved the falsehood of this assertion. A similar opinion has been lately revived, but the period of security extended to five or six years. Neither analogy nor experience justifies such an idea, and the history of casual Cow-Pock, fully refutes the allegation, as numerous cases are on record of persons, after having casual Cow-Pock, resisting during a long life the Small-Pox, under every circumstance of exposure, inoculation, &c. Besides, had the preventive powers of Cow-Pock not been permanent, it is but reasonable to suppose that many of the above 12,065 must, under the existing circumstances of exposure have taken the Small-Pox. Above twenty children who were vaccinated five or six years ago, have lately, by order of the Directors, been submitted to variolous inoculation, but without the effect of producing Small-Pox. Similar experiments have been instituted, under the direction of other practitioners, with the like result. Nineteen children who had the Cow-Pock eight and nine years ago, have been lately inoculated with Small-Pox matter, at the Foundling

 Hospital

Hospital, under the inspection of Mr. Stewart, Surgeon-General, and Mr. Creighton, Surgeon of the Hospital, but with no other effect than local inflammation. In a letter just received from Mr. Bryce, of Edinburgh, he observes—" I have lately finished an experiment of inoculating about twenty children with the Small-Pox, who were vaccinated from eight years to five months. The result is most satisfactory, and shews clearly that a pustule with surrounding inflammation is as readily produced five months after vaccination, as at the end of eight years, consequently that the security is as complete at the latter period as the former."

The following extract from the report of the Small-Pox Hospital, London, should be recorded:—" Eleven thousand eight hundred patients, and upwards, have been vaccinated, of which number *twenty-five hundred* were afterwards *proved* to be secured from the natural Small-Pox, by receiving a further inoculation with Small-Pox matter, which took *no effect*. A number *amply sufficient to satisfy the public mind*, of the *security* and *success* of the new practice of Vaccination." *December*, 1802. So great a number submitted to the test of variolous inoculation, and exposed in a Hospital full of Small-Pox infection *without effect*, should of itself convince every reasonable mind of the efficacy of vaccination.—*Vid. Mr. Charles Murray's Answer to Mr. Highmore, p.* 37.

A report having been lately circulated, that Dr. *Jenner* himself was beginning to entertain some doubt of the efficacy of his discovery, the Directors thought it expedient to direct their Secretary to write to him, and to lay his answer upon the subject before the Public.

" *Dear Sir*,

"Your obliging Letter of the 3d Inst. enclosing the *Annual Report of the Cow Pock Institution, in Dublin*, has just reached me. The former Letter you allude to has not yet been delivered. It is with the greatest pleasure I perceive the rapid increase of vaccination in your Metropolis, and the uninterrupted success that has attended the practice, at once a proof of the zeal, industry and attention of the medical officers, for which I beg leave to make my most grateful acknowledgments.

And now, Sir, a few remarks on the very extraordinary communication you have made to me respecting Lady C———. It has been one of the usual devices of the enemies of vaccination, almost from the time of my first making it known, to represent me as having lost my confidence of its prophylactic powers, or at least, that I was wavering on the subject. Can I, who, with the aid of my nephews, have vaccinated a number of persons little short of 30,000, without one single instance of accident or of failure, that ever reached my ears, for a moment entertain such an absurd idea? Or could I have ever thought of inoculating for the Small Pox, while I hold that practice in abhorrence, and condemn it both publicly and privately? Believe me the whole story you relate to me, is an entire fiction, without the faintest shadow of foundation. Never from the commencement of my experiments to the present hour, have I used a particle of variolous matter, except for the purpose of putting some of those to a test, on whom I made my first trials. For some years past, I have relied wholly on the vaccine lymph, for *testing* those on on whom any material irregularity appeared in the progress of the pustule.

Believe me, &c.

*Berkeley, February,* 19, 1809. EDWARD JENNER."

While the Directors, with such weight of evidence in its favour, feel themselves warranted in continuing to recommend vaccination as a preventative of Small Pox, they cannot but regret that in a few cases it has been difficult to determine whether a patient has had the disease *constitutionally* or *locally.* They, however, confidently hope that by pursuing Mr. Bryce's test, and by increased attention to the progress of the disease, practitioners will be enabled to surmount the only objection to practice which tends to preserve more than 30,000 lives annually, in the British Isles.

Mr. *Bryce* proposes that, a second inoculation be performed about the sixth day after the first, the vesicle produced by this second inoculation is accelerated in its progress, so as to arrive at maturity, and again fade, at nearly the same time as the affection arising from the first inoculation. Mr. B. considers the acceleration of the second inoculation to be the effect of the constitutional affection produced by the first, and therefore if it shall be found that no such acceleration takes place, but that the second inoculation proceeds by a slow progress through all the stages, it is to be concluded, that no constitutional action takes place from the first insertion of the virus; and when this is the case, the second inoculation must be regarded as a primary affection, and a third puncture made according to the plan laid down for conducting the second inoculation, and thus, (he says) we may go on until the proper test be obtained ; or until we be satisfied that the constitution completely resists the action of Cow-Pox.

Although Small-Pox is by no means exterminated from Dublin, among the poor, yet the general substitution of vaccine for variolous inoculation, has considerably diminished the number of patients brought to the Hospitals and Dispensaries for advice. In the upper ranks of society death from Small-Pox is unheard of, and the most extensive practitioners acknowledge that a case of Small-Pox in private practice is a very rare occurrence. And although the re-introduction of Small-Pox into Society would add greatly to the emoluments both of Physic and Surgery, there is no liberal man in either profession who would not sincerely deplore such a calamity.

Signed by Order,

*January* 10, 1810. S. B. LABATT, Sec.

*FOUND-*

*FOUNDLING HOSPITAL*, DUBLIN, *4th* JAN. 1810.

*The following REPORT having been laid before the GOVERNORS of the FOUNDLING HOSPITAL, and appearing to be highly satisfactory: ORDERED, That Three Thousand Copies thereof be printed, for the Purpose of their being circulated as generally throughout the United Kingdom as possible. By Order,*

*A. BAILIE, Register.*

---

As some persons have lately attempted to prejudice the minds of the public, by representing Vaccine Inoculation as a doubtful security against small-pox, limiting its influence to a certain period, and wishing us to believe, that its preventive powers diminish in proportion to the distance of time from Inoculation. I have, therefore, at the request of the Right Honourable and Honourable the Governors of the Foundling Hospital, instituted such experiments as enable me (a second time) to congratulate the public on their successful event.

From my situation, as surgeon to the Foundling Hospital, I have had it fully in my power to select such cases as had been faithfully recorded by me to have undergone vaccination at the earliest period of Cow-Pock Inoculation in this city, and such have been approved of by those gentlemen who have honoured me with their presence, to witness and subscribe their names to the progress and event of the following experiment on nineteen children chosen for the purpose, who were divided into two classes. The first nine, comprehending those who, in a state of infancy, were vaccinated by me between the 30th December, 1800, and 3d July, 1801, now more than eight years. These were again inoculated with Small-Pox infection by George Stewart, esq. Surgeon-General, on the 24th July, 1804, (and witnessed by several gentlemen of the first respectability in their profession) in like manner to disprove the assertions of Mr. Goldson, as may be seen in the twelfth volume of the Medical and Physical Journal, and with the most complete success—all having resisted the Small-Pox, although exposed to it in every way possible. These nine children, with ten others, who were also vaccinated by me in a state of infancy, from 15th July, 1801, to 30th of August, 1802, upwards of seven years, were again submitted to Small-Pox Inoculation on Friday, 22d December last. The infection taken from a child of Mr. Stafford's, No. 7, Hanbury-lane, in confluent Small-Pox, and the matter inserted in two places in the arm of each child, in a fluid state, and in the greatest quantity. In every instance, the punctures in the arm of each child, from the third day, inflamed, and continued until the seventh, when the inflammation gradually subsided, as certified by Mr. Stewart, and marked in a table, which, in another publication, will be more fully expressed; which circumstance has proved the activity of the Small-Pox matter inserted, and which must have affected the constitution, was it in the least susceptible of the disease. Fourteen days have now elapsed, the inflammation of the punctures is entirely gone, and never was attended with the slightest *fever*, *sickness*, or *eruption*.

In corroboration of the above facts, conducted with every degree of accuracy, accuracy, and which cannot admit of the smallest doubt on the minds of those gentlemen who have witnessed them, and hereunto subscribed their names; I can safely assert, that I have submitted upwards of five hundred infants and children, vaccinated by me at this Institution, and at the Dispensary for infant poor and Cow-Pock Inoculation, as established in the year 1800, to a like experiment, and with the same result in every instance.

DUBLIN, MERRION-SQUARE, WEST, J. CREIGHTON.

JANUARY 4, 1810.

*George Stewart*, *Ralph S. Obre*, *Will. Hartigan*, *Gustavus Hume*, *A. Colles*, *Phil. Crampton*, *S. Wilmott*. } Members of the Royal College of Surgeons in Ireland.

*Edmund Connell*, *William Dillon*, *Samuel Bell*, *J. Mc. Creight*. } Apothecaries.

---

### *Fourth Annual Report of the Nottingham Vaccine Institution.*

THE preceding reports have not only traced the progress of the Institution from the establishment in 1805, but have presented to the public authentic and incontrovertible documents in support of vaccine inoculation; and the directors fully confirmed in the opinion of its efficacy, again, in this their fourth report, conscientiously recommend it as a safe and certain practice to eradicate that destructive pestilence the small-pox. Four children were in the months of May, June, and July last, attacked with this highly infectious disease, in consequence only of their inhabiting houses in which the contagion had remained since the epidemic of last year; this most clearly shews the necessity not only of cleaning all the furniture, but of lime-washing the apartments of every house in which the small-pox has appeared, and more particularly when about to be occupied by a fresh tenant. The disease was in another instance introduced by a stranger from London, but in this, as well as in the former cases, it was prevented from spreading, by vaccinating all the individuals exposed to its baneful influence; thus by the powerful and salutary agency of vaccine inoculation the small pox was confined to the subjects it first attacked.

The vaccinations up to the last report, in October 1809, were 3004; to these may now be added 307. This is apparently a small number, but when we reflect on the great prevalence of scarlet fever during the last six months, it will be obvious, that the practice must have been much restricted, as it is a principle of this Institution not to inoculate children either affected with or suffering from fever, except under very particular circumstances, which are always noted in the register.

The following then is the present state of the Register:

| | |
|---|---|
| 2787 | Satisfactory or perfect vaccinations, |
| 332 | Doubtful, or unsatisfactory vaccinations, |
| 173 | Blank, or those where the inoculation did not take effect, |
| 19 | Under progress of vaccination. |
| 3311 | Total of persons inoculated for cow-pock. |

The division of cases into four heads has been too often explained to require any comment, further than our decided approval of the distinction which has been thus drawn from the first, and our conviction that it not only facilitates, but gives precision to the practice.

This Institution, founded and supported by benevolence, can only be efficient whilst it receives the protection of the public; it requires but little, but that little is necessary to its existence, yet the good effected by such small means is beyond calculation; and the benevolent, who have hitherto given this protecting shield to the poor, will, we are fully persuaded, still advance an arm for its support.

By order of the Directors,

GEORGE COLDHAM, Secretary.

*Nottingham, Oct.* 18, 1810.

---

(From the London Medical Review for October 1810.)

*Effect of a Ligature on the Iliac Artery.*

The remarkable effect which follows the application of a ligature to an artery, and its subsequent removal, as mentioned by Dr. Jones in his Treatise on Hæmorrhage, has lately been exemplified in the human subject. The particulars of the case, as far as we have been able to ascertain them, are as follows. A man underwent the operation of castration; the arteries were separately tied; in a few hours afterwards one of the ligatures came away, and a profuse hæmorrhage followed. Various means were used to restrain the bleeding, but it recurred at different periods for several weeks. The spermatic chord was followed by a dilatation of the abdominal ring, and pressure by means of a spring truss employed with as much force as could be borne. At the expiration of some weeks from the application of the truss, a violent hæmorrhage took place from a very large vessel passing from the thigh in a direction upwards and inwards. In this emergency the external iliac artery was exposed by an incision. A ligature was placed around it, and when drawn tight the hæmorrhage ceased. In a few minutes the ligature was removed, and the circulation was observed to be distinctly carried on in the artery below the part where the ligature had been applied. A slight hæmorrhage took place afterwards, but the wound did well, and had granulated and nearly healed, when the patient was destroyed by a pulmonic disease. Upon dissection, the external iliac artery was found completely obliterated for the space of three inches, extending from the origin of the internal iliac to the point where the vessel passes under Poupart's ligament. It should be observed that the vein in the same situation was likewise obliterated by a very firm plug of coagulum. It may be a question in this case, since the coagulum was extensive and the vein participated in the obliteration, in what degree the pressure applied influenced the appearances.

---

*Dilatation of the Male Urethra.*

A boy aged four years, laboured under suppression of urine. He had always borne the appearance of a cripple: his lower extremities were were small and crooked. On attempting to introduce a catheter, it was obstructed by a calculus, lying in the membranous part of the urethra. The surgeon, whose name we have no authority to mention, with some difficulty introduced a very small one, and drew off his urine. He then introduced a sponge tent into the urethra, and allowed it to remain there until the next day, which operation he repeated, increasing the size of the tent daily, for about a fortnight, at which time the passage was so much dilated as nearly to admit of the introduction of the little finger. One morning, in making an effort to void his urine, a calculus of the size of a large nutmeg was discharged; and on the following day, two more of the same size. In a short time the boy gained strength and the perfect use of his limbs, as well as the power of retaining his urine. The tents employed were in the form of bougies, which were made by rolling together long strips of well prepared sponge. The calculi are of an irregular wedge-like shape, with their sides flattenened and rendered smooth by contact, weighing, on an average, from 60 to 70 grs. each. We consider this case as highly important, and very creditable to the gentleman who conducted it.

---

*Longevity and Hybernation of the Testudo Græca.*

"THERE is now living in the garden belonging to the Bishop's Palace, at Peterboro', a land *Tortoise*, which is ascertained to have lived there 200 years. The upper shell is near 14 inches long, 9 broad, and the weight of the animal is 20 pounds. It has lost the sight of one eye, the other is bright and lively: the inside of the mouth as well as the tongue, is a full pink colour. The legs, feet, and head are covered with scales. In the early part of the summer, it in general feeds upon lettuce; but gathers up fallen fruit, especially goosberries. Towards Michaelmas, and sometimes earlier, it buries itself in the earth, where it remains till the following spring. In a few days after it has made this annual descent, by finding the depth with a stick, a tolerably accurate judgment may be formed of the mildness or severity of the ensuing winter."

There are circumstances in this account of great interest to the naturalist, especially the extreme longevity of this animal. We may be allowed, however, to doubt the accuracy of the relation that states it to have existed upwards of 200 years; and to hope that some of our correspondents at Peterboro', or the vicinity, will take the trouble to ascertain the real fact.

We suppose this animal to be the *Testudo Græca*, a creature extremely tenacious of life, suffering the most violent mutilations, and extraordinary privations, without being destroyed. This species of tortoise has been considered as arriving at a very extended longevity. *M. Cetti* saw one in Sardinia which had lived sixty years in one house, where it was considered as an old domestic. Its power of surviving the loss of organs, which have been considered as essential to the continuance of life in other animals, has been ascertained by a series of experiments made by Sig. Redi. In the month of November he made a large opening in the skull of a *Testudo Græca*, and removed the whole substance of the brain, cleansing out the cavity with great care. The eyes of the animal instantly closed, and never afterwards opened; but being set at

 liberty, liberty, it continued to walk about as well as before the brain was taken away. In three days the wound was covered with new skin, and the tortoise lived, and continued all its ordinary motions, till the middle of May, nearly six months after it had been deprived of its brain. After death, only a small black clot of dry blood was found in the cavity, from which the brain had been taken out. He repeated this experiment on several tortoises, both land, fresh water, and even the sea species, and they all continued to live, for longer or shorter times, after losing their brain. Another remarkable property of the genus *Testudo* is, that the individuals of it can live a long time without food. *Gerard Blasius* kept a *Testudo Græca* ten months without food, and then it died, not from inanition, but from excessive cold. The intestines of this were found full of excrementitious matter.

The genus *Testudo*, in some of its species, is common to all the warm and temperate countries of the old world. It is found in Southern Europe, in Amboyna, Ceylon, Japan, Bourbon, and India; also in the desert parts of Africa. There is some doubt whether the land tortoise is indigenous to America, or has been transported thither from the old world.

---

The Middlesex Hospital, which had long laboured under great difficulties, for want of Funds, has lately met with extraordinary support, from the liberality of the Public, and of private Individuals. By the Benefit concert, which was given at the Opera House about eighteen months ago, through the kindness of Mr. Taylor, it cleared upwards of two thousand pounds; an unknown Gentleman has, since that time, called at the Hospital, and left one thousand three hundred and twenty pounds with the Secretary, and Mrs. Stafford, a Lady, residing in Hill-street, Berkeley-square, has within the last six weeks left by will five thousand pounds to the Cancer Fund, and one third of her residuary property, to the general purposes of that Hospital: this third, it is supposed, will produce nearly eight thousand pounds; it is to be hoped, therefore, that this large and well constructed Hospital, will soon be in a condition to give relief to all the poor objects that may want its charitable assistance; of late years great numbers have been denied admittance on account of the poverty of the Institution.

---

Dr. Clough, Physician, Man-midwife to the St. Mary-le-bone General Dispensary, will, on Monday the 14th of January, 1811, at Ten in the Morning, commence his Second Winter Course of Lectures on the Science of Midwifery, including the Diseases of Women and Children, at his Lecture-room, 68, Berner's-street, where Syllabusses and Prospectuses may be had. N. B. His evening course is continued, as usual, on Tuesdays, Thursdays, and Saturdays, at the hour of Seven, during the Winter.

---

Dr. George Rees is preparing for the press a new edition of his book on Disorders of the Stomach, in which many additional cases and important observations will be introduced.

A Treatise

A Treatise on some practical Points relating to the Diseases of the Eye, by the late J. C. SAUNDERS, esq. is in the press. It will be illustrated by coloured engravings, and contain a short account of the author's life, with an engraving from a portrait by Devis.

---

Dr. HOOPER will, in a few days, publish the first fasciculus of his long promised Anatomical Atlas.

